# Cancer incidence in Iran in 2016: A study based on the Iranian National Cancer Registry

**DOI:** 10.1002/cnr2.1967

**Published:** 2023-12-26

**Authors:** Fatemeh Hadavandsiri, Leila Allahqoli, Yekta Rahimi, Hamid Salehiniya, Ehsan Ghazanfari Savadkoohi, Mohammad Esmaeil Akbari

**Affiliations:** ^1^ Cancer Research Center Shahid Beheshti University of Medical Sciences Tehran Iran; ^2^ Ministry of Health and Medical Education Tehran Iran; ^3^ Student Research Committee, School of Public Health and Safety Shahid Beheshti University of Medical Sciences Tehran Iran; ^4^ Social Determinants of Health Research Center Birjand University of Medical Sciences Birjand Iran; ^5^ Department of Health Information Technology and Management, School of Allied Medical Sciences Shahid Beheshti University of Medical Sciences Tehran Iran

**Keywords:** cancer, cancer registry, incidence, Iran

## Abstract

**Background:**

Cancer poses an escalating public health challenge, necessitating a comprehensive understanding of cancer incidence to formulate effective control strategies.

**Aims:**

This study aims to present a comprehensive overview of cancer incidence in Iran, utilizing data from the Iranian National Population‐based Cancer Registry (INPCR) for the year 2016.

**Methods:**

The study employed INPCR data to compute crude and age‐standardized incidence rates (ASR) per 100 000 for the most common cancers among men and women across Iran's 31 provinces. Data analysis utilized Excel (2019) and STATA 14.

**Results:**

In 2016, 124 833 new cancer cases were registered, with 65 495 (52.90%) occurring in men and 58 312 (47.10%) in women. ASRs for all cancers in the total population were 177.48, with specific rates for men and women at 192.96 and 162.33, respectively. The five most common cancers in men were prostate (23.25), stomach (21.56), colon (19.30), bladder (16.20), and lung (13.15). Among women, the leading cancers were breast (40.60), colon (14.64), thyroid (10.84), stomach (10.25), and lung (5.63). West Azarbaijan had the highest incidence among men, while Yazd topped the list for women. Age‐specific incidence rates revealed peaks in the 67–74 age group for men and the 40–50 age group for women.

**Conclusion:**

This study affirms that while Iran exhibits a lower cancer incidence compared to global averages, there has been a temporal increase. Disparities in ASR exist across sexes and provinces, with shifts in the ranking of common cancers by sex compared to previous reports.

## 1 INTRODUCTION

Cancer represents a global health concern,^1^ with 17.2 million cases reported worldwide in 2016.^2^ In Iran, it ranks as the second most prevalent chronic non‐communicable disease and the third leading cause of death after heart disease, accidents, and natural phenomena.^3^ The ASR for all cancer types in men and women, excluding non‐melanoma cancer, was 165.0 and 139.0 per 100 000, respectively.^4^ Estimated cancer death rates were 65 and 41.1 per 100 000 for men and women, respectively.^5^

Projections indicate a potential increase in global cancer cases to 28 million by 2040,^6^ with a 43% rise in new cases in Iran from 2016 to 2025.^7^ A cancer projection for Iran suggests a 2.17 times increase in ASR for all cancer sites by 2035.^8^

A 2014 survey of cancer registries in Iran highlighted stomach, prostate, and colorectal cancers as prevalent among men, and breast, colorectal, and stomach cancers among women. However, registry coverage varied widely across states and provinces.^9^ Cancer incidence in Iran exhibited a rise from 2008 to 2014,^8^, ^9^ characterized by significant heterogeneity in cancer type, province, and sex.

Iran, a Middle Eastern developing nation with a population of 85 million as of 2020,^10^ faces various risk factors linked to cancer precursors.^11^, ^12^, ^13^ Recent lifestyle and environmental changes may influence the epidemiological pattern of cancer.^14^, ^15^, ^16^ With an aging population, increased life expectancy, and notable risk factors, cancer incidence is rising globally, especially in West Asian populations. This situation is critical for developing countries like Iran, where cancer management is a significant concern. Consequently, a quantitative evaluation of cancer‐related epidemiological metrics is crucial for devising effective prevention and management strategies.^9^, ^17^

Cancer registries play a pivotal role as information sources for cancer‐related epidemiology.^18^ They collect, store, and manage data critical for cancer surveillance, research, and the planning and evaluation of prevention and control interventions.^14^ This study aims to unveil the latest cancer incidence data, providing ASR for all cancer categories across Iran's 31 provinces in 2016. The inclusive approach covers both genders and all age groups, aiming to inform effective strategies for cancer prevention and management in Iran.

## 2 MATERIALS AND METHODS

### 2.1 Study population and data source

The study utilized data from the Iran Statistics Center website^19^ to represent the entire Iranian population. According to the 2016 National Population and Housing Census, Iran's total population across 31 provinces was 79 926 270, with 40 498 442 men and 39 427 828 women.

To analyze cancer incidence in Iran, the study relied on data from the Iranian National Population‐based Cancer Registry (INPCR). Managed by all 60 medical universities under the Iranian Ministry of Health and Medical Education (MOHME), the INPCR covers 31 provinces. The 2016 data collection adhered to International Agency for Research on Cancer (IARC) and International Association of Cancer Registries (IACR) standard protocols. Trained staff in 60 universities facilitated the cancer registry, utilizing pathology reports and clinical/paraclinical data from hospitals. Patient residence was the basis for registration to prevent duplicate cases in different provinces. However, around 30 679 patients lacking a national ID prompted the use of first name, surname, father name, date of birth, and gender for data pooling. Information gathered included personal details and tumor encoding using the International Classification of Diseases for Oncology, Third Edition (ICD‐O‐3).^20^

After data collection, each university's cancer registry staff performed quality control and data processing, including internal consistency checks and duplicate record verification.

### 2.2 Statistical analysis

Data analysis employed Excel (2019) and STATA 14, calculating frequency, percentage, crude and age‐standardized incidence rates (ASR) for all cancer types and the 10 major cancer types in both sexes across 31 provinces and nationally. Median and interquartile range (IQR) of age were also computed.

The ASR, expressed per 100 000 population and standardized to the Segi‐Doll world population, utilized the direct method. This involved adding products of age‐specific rates and the number of persons in the same age subgroup of the standard population, divided by the sum of weights of the standard population.

## 3 RESULTS

According to the Iranian National Cancer Registry (INPCR) in 2016, Iran recorded 124 833 new cancer cases, with 52.90% in men and 47.10% in women. The median age (IQR) was 64 (75–53) years for men and 57 (69–44) years for women. The crude rate for all cancers in the total population was 154.90, with an ASR of 177.48 (per 100 000 people). ASRs for all cancers were 192.96 for men and 162.33 for women. ASRs for all cancers, excluding non‐melanoma skin cancers, were 170.50 for men and 148.43 for women. Table 1 presents estimated new cases, crude and ASR for invasive cancers by sex and cancer type.

**TABLE 1:** Estimated new cases and age standard rate by sex and cancer type.

Cancer groups	Estimated new cases		Estimated crude rate and ASR					
	Men	Women	Total crude rate	Total ASR	Crude rate in men	ASR in men	Crude rate in women	ASR in women
All sites	65 495 (52.90)	58 312 (47.10)	154.90	177.48	161.72	192.96	147.90	162.33
Oral cavity and pharynx	1297 (57.03)	977 (42.97)	2.85	3.22	3.20	3.65	2.48	2.78
Tongue	356 (52.98)	316 (47.02)	0.84	0.96	0.88	1	0.80	0.92
Mouth	119 (52.42)	108 (47.58)	0.28	0.33	0.29	0.34	0.27	0.32
Pharynx	40 (51.95)	37 (48.05)	0.10	0.11	0.10	0.11	0.09	0.11
Other oral cavities	782 (60.25)	516 (39.75)	1.62	1.82	1.93	2.19	1.31	1.44
Digestive system	19 755 (60.28)	13 015 (39.72)	41.00	48.83	48.78	59.2	33.01	38.53
Esophagus	1977 (55.59)	1579 (44.41)	4.45	5.4	4.88	5.98	4.00	4.81
Stomach	7104 (67.36)	3442 (32.64)	13.19	15.89	17.54	21.56	8.73	10.25
Small intestine	449 (59.39)	307 (40.61)	0.95	1.1	1.11	1.29	0.78	0.9
Colon	4440 (55.53)	3556 (44.47)	10.00	11.73	10.96	13.1	9.02	10.37
Rectum	1544 (59.45)	1053 (40.55)	3.25	3.77	3.18	4.54	2.67	3.01
Anus, anal canal, and anorectum	95 (60.90)	61 (39.10)	0.19	0.23	0.23	0.29	0.15	0.18
Liver and intrahepatic bile duct	1522 (60.01)	1014 (39.99)	3.17	3.79	3.76	4.55	2.57	3.03
Gallbladder and other biliary	417 (48.94)	435 (51.06)	1.06	1.29	1.03	1.25	1.10	1.32
Pancreas	1353 (60.50)	883 (39.50)	2.80	3.39	3.34	4.12	2.24	2.67
Other digestive organs	854 (55.49)	685 (44.51)	1.92	2.25	2.11	2.51	1.74	1.99
Respiratory system	6452 (71.90)	2521 (28.10)	11.23	13.33	15.93	19.3	6.39	7.41
Larynx	1506 (86.70)	231 (13.30)	2.17	2.58	3.72	4.49	0.59	0.68
Lung and bronchus	4351 (69.75)	1887 (30.25)	7.80	9.37	10.74	13.15	4.79	5.63
Other respiratory organs	595 (59.61)	403 (40.39)	1.25	1.38	1.47	1.66	1.02	1.11
Bones and joints	672 (57.14)	504 (42.86)	1.47	1.58	1.66	1.8	1.28	1.37
Soft tissue	877 (53.74)	755 (46.26)	2.04	2.17	2.17	2.34	1.91	2
Skin	7434 (61.25)	4702 (38.75)	15.18	18.18	18.36	22.45	11.93	13.91
Melanoma of the skin	‐	‐	‐	‐	‐	‐	‐	‐
Other nonepithelial skin	7434 (61.25)	4702 (38.75)	15.18	18.18	18.36	22.45	11.93	13.91
Breast cancer	292 (1.87)	15 333 (98.13)	19.55	20.68	0.72	0.83	38.89	40.60
Genital system	8247 (61.84)	5089 (38.16)	16.69	19.66	20.37	25.25	12.91	14.08
Uterine cervix	‐	806 (15.84)		‐		‐	2.04	2.16
Uterine corpus	‐	1602 (31.48)		‐		‐	4.06	4.54
Ovary	‐	1923 (37.79)		‐		‐	4.88	5.27
Vulva	‐	59 (1.16)		‐		‐	0.15	0.18
Vagina and other genital, women	‐	699 (13.73)		‐		‐	1.77	1.93
Prostate	7383 (89.52)	‐		‐	18.23	23.25		‐
Testis	817 (9.91)	‐		‐	2.01	1.86		‐
Penis and other genital, men	47 (0.57)	‐		‐	0.12	0.14		‐
Urinary system	7024 (76.82)	2119 (23.18)	11.44	13.54	17.34	20.96	5.37	6.19
Urinary bladder	5378 (82.20)	1165 (17.80)	8.19	9.81	13.28	16.2	2.95	3.48
Kidney and renal pelvis	1526 (62.36)	921 (37.64)	3.06	3.50	3.77	4.40	2.34	2.60
Ureter and other urinary organs	120 (78.43)	33 (21.57)	0.19	0.23	0.30	0.36	0.08	0.10
Eye and orbit	181 (56.74)	138 (43.26)	0.40	0.44	0.45	0.50	0.35	0.39
Brain and other nervous systems	2639 (57.28)	1968 (42.72)	5.76	6.24	6.52	7.10	4.99	5.38
Endocrine system	1284 (21.78)	4611 (78.22)	7.38	7.22	3.17	3.30	11.69	11.20
Thyroid	1111 (19.88)	4477 (80.12)	6.99	6.80	2.74	2.83	11.35	10.84
Other endocrine	173 (56.35)	134 (43.65)	0.38	0.42	0.43	0.48	0.34	0.36
Lymphoma	208 (57.62)	153 (42.38)	0.45	0.50	0.51	0.58	0.39	0.43
Hodgkin lymphoma	35 (61.40)	22 (38.60)	0.07	0.08	0.08	0.09	0.05	0.06
Non‐Hodgkin lymphoma	173 (56.91)	131 (43.09)	0.38	0.43	0.43	0.49	0.33	0.37
Myeloma	845 (60.10)	561 (39.90)	1.76	2.11	2.08	2.53	1.42	1.68
Leukemia	2229 (61.52)	1394 (38.48)	4.53	5.01	5.50	6.15	3.53	3.86
Acute lymphocytic leukemia	675 (59.26)	464 (40.74)	1.42	1.52	1.66	1.76	1.18	1.27
Chronic lymphocytic leukemia	536 (67.76)	255 (32.24)	0.99	1.2	1.32	1.63	0.65	0.77
Acute myeloid leukemia	720 (59.11)	498 (40.89)	1.52	1.64	1.78	1.95	1.26	1.34
Chronic myeloid leukemia	181 (56.21)	141 (43.79)	0.40	0.43	0.45	0.49	0.36	0.38
Other leukemia	117 (76.47)	36 (23.53)	0.19	0.21	0.29	0.32	0.09	0.10
Other and unspecified primary sites	1299 (58.43)	924 (41.57)	2.78	3.17	3.21	3.71	2.34	2.63

Table 2 highlights the number, percentage, crude rate, and ASR per 100 000 for the 10 most common cancers in both sexes in Iran. Prostate, stomach, colorectal, bladder, and lung were the five most common cancers in men, while breast, colorectal, thyroid, stomach, and lung were predominant in women.

**TABLE 2:** Ten leading and most common cancer types for the new cancer cases by sex, Iran, 2016.

		Number	Percent	Crude rate	ASR
Men	Prostate	7383	18.17	18.23	23.25
	Stomach	7104	17.48	17.54	21.56
	Colorectal	6546	16.11	16.16	19.30
	Bladder	5378	13.24	13.28	16.20
	Lung	4351	10.71	10.74	13.15
	Brain	2639	6.50	6.52	7.10
	Leukemia	2229	5.48	5.50	6.15
	Esophagus	1977	4.86	4.88	5.98
	Liver	1522	3.75	3.76	4.55
	Larynx	1506	3.70	3.72	4.49
	All sites	65 495	100	161.72	192.96
	All sites except C44^a^	58 061	‐	143.36	170.50
Women	Breast	15 333	40.13	38.89	40.60
	Colorectal	5045	13.20	12.80	14.64
	Thyroid	4477	11.72	11.35	10.84
	Stomach	3442	9.01	8.73	10.25
	Lung	1887	4.94	4.79	5.63
	Brain	1968	5.15	4.99	5.38
	Ovary	1923	5.03	4.88	5.27
	Esophagus	1579	4.13	4.00	4.81
	Leukemia	1394	3.65	3.53	3.86
	Bladder	1165	3.04	2.95	3.48
	All sites	58 312	100	147.90	162.33
	All sites except C44^a^	53 611	‐	135.97	148.42

^a^ Non‐melanoma skin cancer.

Tables 3 and 4 provide information on the 10 most common cancers in both sexes across 31 provinces and 4 regions. The study identified regional variations, with North/Northwest and Central Iran having high ASRs for both men and women. West Azarbaijan and Sistan and Baluchistan recorded the highest and lowest ASRs for all sites except non‐melanoma skin cancer in men, respectively. In women, Yazd and Sistan and Baluchistan showed the highest and lowest ASRs for the same category.

**TABLE 3:** Age‐standardized incidence rates (per 100 000) of the 10 most common cancers and all cancers in different regions and provinces of Iran, 2016 (men).

Region	Province	Total population	All cases	All sites ASR	All sites but C44^a^	Prostate	Stomach	Colorectal	Bladder	Lung	Brain	Leukemia	Esophagus	Liver	Larynx
North/North West	Ardabil	635 687	1311	214.11	193.27	16.4	43.35	25.73	10.85	20.93	9.62	5.03	13.05	5.53	3.38
	Golestan	938 327	2520	210.66	198.45	18.57	27.85	22.05	12.94	23.06	8.62	8.24	15.33	5.38	4.23
	Guilan	1 267 597	1877	171.86	155.77	17.33	25.28	21.31	14.03	14.13	7.10	4.14	4.60	3.53	4.11
	Mazandaran	1 653 998	1894	188.73	172.01	24.58	26.93	19.89	12.37	14.15	3.21	5.69	7.64	2.32	4.31
	North Khorasan	433 633	376	181.03	158.52	11.37	34.85	10.14	9.87	14.59	8.53	1.40	13.45	4.60	9.72
	East Azarbayjan	1 989 400	2171	215.61	192.43	23.04	31.20	24.16	21.93	13.12	5.98	8.94	9.28	3.76	3.78
	Kurdistan	712 776	668	174.00	159.57	13.43	25.23	12.30	8.01	17.30	6.72	4.34	9.31	6.94	4.17
	West Azarbayjan	1 658 319	1877	231.95	206.70	19.58	36.35	17.38	18.93	21.52	10.33	7.20	12.98	6.49	6.00
	Zanjan	534 849	326	119.57	102.6	9.51	19.05	11.66	7.33	9.61	3.39	4.34	7.27	2.14	2.36
West	Hamedan	880 318	878	188.85	159.56	27.38	17.68	13.20	14.79	14.36	5.84	7.70	3.57	6.58	4.96
	Ilam	295 199	596	184.73	160.02	16.54	22.22	24.63	9.37	13.23	8.15	6.99	11.17	5.59	3.11
	Kermanshah	988 015	747	141.08	124.82	13.36	13.80	12.06	16.14	11.44	5.48	3.73	4.42	5.19	3.23
	Khuzestan	2 388 674	1599	179.67	160.03	24.75	14.54	15.13	15.68	13.54	7.05	3.02	2.19	5.50	4.51
	Lorestan	892 889	477	127.05	108.16	9.08	15.35	7.70	10.86	7.10	5.28	2.35	4.15	2.61	4.11
Center	Alborz	1 376 335	1272	181.95	165.96	25.07	23.54	22.11	14.07	12.77	7.53	3.91	3.85	4.13	4.60
	Chaharmahal‐bakhteiari	482 356	285	150.50	127.665	14.22	15.19	13.22	17.53	5.30	5.95	3.03	1.40	3.22	2.21
	Qazvin	650 499	580	177.10	158.67	10.07	29.18	17.43	11.58	14.15	9.97	5.40	6.64	8.86	2.87
	Isfahan	2 599 477	2784	216.87	184.97	30.08	12.88	19.82	18.38	13.17	8.60	13.55	2.96	5.11	3.38
	Qom	658 540	557	212.04	193.83	20.56	23.84	23.39	17.14	10.56	5.41	0.36	8.86	7.42	2.01
	Kerman	1 617 688	1369	197.20	175.40	18.27	16.23	13.36	23.22	19.08	8.72	6.08	4.11	7.34	8.23
	Kohkilooye‐Boyerahmad	361 386	167	130.75	107.91	10.60	19.40	10.10	9.87	3.19	4.57	5.99	2.38	4.37	1.51
	Markazi	725 751	790	172.07	153.66	19.80	22.86	17.19	15.25	9.40	10.61	1.23	5.38	8.12	5.55
	Tehran	6 673 672	6151	234.46	147.67	23.29	16.50	21.85	12.78	10.19	5.84	3.72	3.71	2.49	4.47
	Semnan	356 656	285	142.25	126.33	15.77	16.09	17.29	15.54	8.09	5.40	1.82	4.30	2.97	5.30
	Yazd	586 013	522	228.26	191.49	28.09	11.75	25.16	17.51	10.53	8.71	10.54	1.81	4.24	5.24
East	Razavi‐Khorasan	3 245 185	3034	204.80	179.53	16.32	31.02	20.91	11.65	14.03	7.98	5.74	10.95	4.61	4.48
	Sistan and Baluchistan	1 401 931	362	92.43	84.48	4.97	11.66	7.09	6.07	4.72	4.24	1.63	3.89	4.43	2.72
	South Khorasan	389 917	316	164.60	144.22	11.41	18.41	14.18	9.39	10.17	9.44	3.11	9.41	5.23	4.53
South	Bushehr	620 722	280	126.25	118.90	16.21	7.23	9.69	11.43	16.93	7.04	5.69	1.92	4.89	2.37
	Fars	2 461 251	1846	167.12	149.84	21.48	12.15	11.08	15.79	10.36	6.97	11.45	2.35	4.65	4.84
	Hormozgan	906 814	374	122.14	113.15	14.91	9.40	10.27	9.74	12.96	4.78	4.16	1.59	3.69	2.50

^a^ Non‐melanoma skin cancer.

**TABLE 4:** Age‐standardized incidence rates (per 100 000) of the 10 most common cancers and all cancers in different regions and provinces of Iran, 2016 (women).

Region	Province	Total population	All cases	All sites ASR	All sites but C44^a^	Breast	Colorectal	Thyroid	Stomach	Lung	Brain	Ovary	Esophagus	Leukemia	Bladder
North/North West	Ardabil	634 733	651	160.40	148.99	24.75	18.13	6.40	26.53	9.25	5.66	3.22	12.06	2.80	3.59
	Golestan	930 492	971	175.34	167.57	40.35	14.07	7.85	12.53	11.98	6.02	5.11	11.64	5.81	2.43
	Guilan	1 263 099	1528	143.63	133.1	38.09	13.47	14.01	10.74	3.45	4.81	5.56	3.88	2.90	3.63
	Mazandaran	1 629 584	1824	163.93	153.36	41.87	15.00	11.03	12.17	4.81	2.91	3.74	5.47	3.87	2.74
	North Khorasan	429 459	311	130.00	119.60	15.72	11.63	5.92	12.86	5.48	7.08	5.13	10.56	1.85	4.04
	East Azarbayjan	1 650 315	1905	186.90	173.11	30.11	15.82	11.78	12.16	5.08	6.80	3.93	7.26	11.70	3.95
	Kurdistan	790 235	509	117.90	108.70	17.26	10.26	4.03	13.12	6.20	5.27	2.17	7.28	2.02	2.10
	West Azerbaijan	1 650 315	1474	161.30	146.08	32.84	14.51	3.65	19.63	6.14	7.23	6.81	11.82	5.60	3.11
	Zanjan	522 612	313	89.53	83.50	18.46	12.74	4.71	9.21	5.39	1.64	2.93	3.43	2.00	2.47
West	Hamedan	857 916	734	134.00	121.03	29.18	12.51	7.93	7.90	5.08	4.74	4.80	3.95	4.64	2.13
	Ilam	284 959	348	197.82	183.55	43.74	22.32	10.01	22.61	8.33	10.27	5.25	9.37	1.55	1.72
	Kermanshah	964 419	810	122.40	114.07	30.19	11.28	9.47	10.14	4.34	3.50	3.84	4.11	2.09	3.31
	Khuzestan	2 321 835	1833	157.84	142.31	45.90	10.29	7.64	7.37	5.31	4.67	4.92	2.54	1.53	4.02
	Lorestan	867 760	492	102.37	90.22	22.17	8.17	5.47	7.77	3.57	3.70	2.06	4.47	1.55	1.86
Center	Alborz	1 336 065	1283	152.50	141.4	45.81	14.68	5.97	11.70	5.96	6.21	4.51	3.58	2.18	3.81
	Chaharmahal‐bakhteiari	465 407	306	117.41	104.89	21.29	7.99	19.24	7.05	2.81	1.50	5.50	1.09	1.80	3.03
	Qazvin	623 262	513	136.60	123.79	26.21	13.66	5.97	12.26	5.15	6.32	4.13	5.24	2.98	2.65
	Isfahan	5 420 373	3212	194.70	175.42	50.61	16.10	20.27	6.75	6.28	5.78	7.04	1.45	7.70	3.19
	Qom	633 743	618	178.20	158.46	43.17	16.70	19.05	9.32	4.68	3.21	6.54	4.24	0.33	3.50
	Kerman	1 547 030	1213	155.50	139.42	35.33	9.11	9.66	7.59	7.95	6.80	5.06	2.74	4.74	3.70
	Kohkilooye‐Boyerahmad	351 666	239	129.17	111.81	18.65	7.59	27.64	9.63	4.85	2.37	2.60	5.06	3.18	3.22
	Markazi	703 724	722	138.69	128.1	37.36	15.03	8.34	8.22	4.62	4.77	6.32	2.61	1.29	3.35
	Tehran	6 593 965	6882	203.21	141.29	46.73	16.89	7.31	8.24	4.35	4.60	6.09	2.71	2.78	2.71
	Semnan	345 704	278	124.52	115.20	33.62	13.26	3.27	6.30	6.53	4.74	3.57	5.85	0.71	2.78
	Yazd	552 520	737	225.40	206.94	46.34	18.45	43.05	7.30	5.37	7.54	8.17	2.70	9.23	4.08
East	Razavi‐Khorasan	3 189 316	3043	178.95	162.10	36.17	15.02	7.86	13.54	7.63	7.24	5.64	8.83	3.46	3.02
	Sistan and Baluchistan	1 373 083	386	74.82	69.94	12.65	7.23	5.65	3.50	1.80	2.00	3.62	4.67	0.66	1.83
	South Khorasan	465 407	264	137.10	121.21	20.03	12.30	8.33	6.58	5.04	5.83	3.74	6.21	2.17	1.85
South	Bushehr	542 678	399	136.10	128.26	32.18	9.16	16.70	4.64	10.48	6.35	3.74	2.16	4.17	3.97
	Fars	2 390 023	2134	144.40	133.20	38.80	8.76	15.72	5.98	4.53	5.86	3.88	1.67	7.01	3.67
	Hormozgan	869 601	463	114.79	107.63	30.07	6.14	5.71	5.21	6.03	3.00	3.88	2.47	2.58	3.31

^a^ Non‐melanoma skin cancer.

**FIGURE 1:**
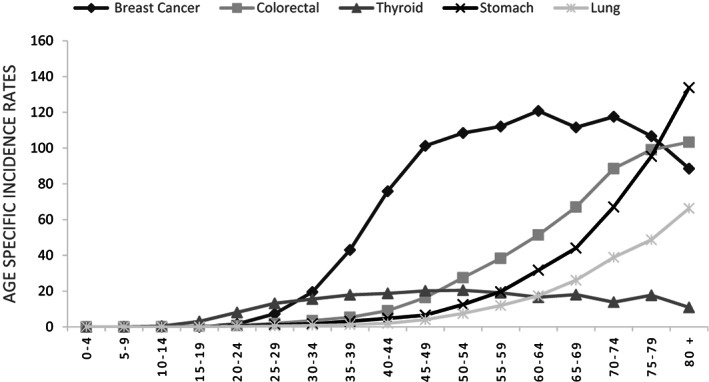
Five most common age‐specific cancer incidence rates (per 100 000) in Iran, 2016 (women).

**FIGURE 2:**
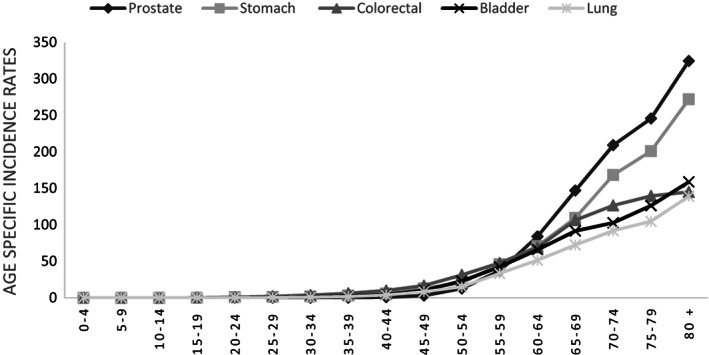
Five most common age‐specific cancer incidence rates (per 100 000) in Iran, 2016 (men).

Age‐specific incidence rates exhibited peaks at 64–74 years for men and 40–50 years for women. The patterns were consistent across sexes, with lower rates in younger age groups and a sharp rise in middle age. Figures 1 and 2 visually represent age‐specific incidence rates for the five most common cancers in both sexes, illustrating this observed pattern.

## 4 DISCUSSION

Cancer, irrespective of human development levels, remains a significant contributor to morbidity and mortality. This study represents the first comprehensive overview of cancer incidence in Iran for 2016. Population‐based cancer registries play a crucial role in informing cancer control policymakers, and our study utilized data from a national population‐based cancer registry in Iran.

Discrepancies between global reports^21^, ^22^ and Iranian national reports highlight the importance of referencing national reports for precise information due to variations in data availability from different provinces. Despite differing rankings between genders, lung, brain, and esophagus cancers consistently occupy the 5th, 6th, and 8th positions, respectively, among the 10 most common cancers. This study delves into the three most common cancers in men and women separately for a more nuanced analysis.

Recent statistics indicate that cancer incidence rates (excluding non‐melanoma skin cancers) in Iranian men (170.50) and women (148.43) are notably lower than global rates for men (206.9) and women (178.1).^23^ However, an anticipated increase in life expectancy and the adoption of a westernized lifestyle suggest a future dramatic rise in cancer incidence rates in Iran.

Stomach cancer, though declining globally, remains a prevalent malignancy, ranking fourth in men and seventh in women worldwide.^24^ In Iran, stomach cancer, especially in men, is still significant. The shift in rankings from the most common cancer in Iranian men in 2014 to the second most common in 2016 may be attributed to men exhibiting earlier onset, supported by our findings.^9^, ^25^ Behavioral factors like smoking and alcohol consumption in men might contribute to the higher lifetime risk of developing stomach cancer.^25^

Prostate cancer, despite being the most common cancer in developed regions,^26^ was the second most common in Iranian men in 2014.^9^ However, our 2016 data reveal a surge in prostate cancer incidence, making it the most common cancer among men. However, a systematic review and meta‐analysis showed that the incidence of prostate cancer in Iran is low compared with other Asian countries such as Turkey and Lebanon.^27^ The increase may be linked to enhanced screening efforts and heightened awareness.

Breast cancer, more prevalent in less developed countries among younger women, maintains its status as the most commonly diagnosed cancer in women worldwide.^28^ Iran, falling within the Eastern Mediterranean Region,^29^ sees a rising incidence of breast cancer.^7^, ^30^, ^31^ The 2016 crude and ASR for breast cancer increased compared to 2014, with an early peak in age‐specific rates observed in younger women.^9^, ^32^

Colorectal cancer, the fourth most common worldwide, shows increasing incidence in developing countries.^33^ In Iran, it retains its ranking as the second most common cancer in women and third in men, consistent with 2014 data.^9^ The geographical pattern aligns for both genders in Iran, although regional differences are reported in the United States and Australia.^34^, ^35^

Thyroid cancer incidence in Iran exhibits a rising trend, particularly among women.^36^, ^37^ This increase may be attributed to improved diagnostic techniques, leading to overdiagnosis.^38^ While Spain reports higher rates due to diagnosis,^39^ Iran's relatively lower rate suggests a lack of thyroid cancer screening programs. Factors like lifestyle, geography, and hormonal influences may contribute to the observed patterns.^40^

Limitations of our research include a lack of information on mortality, survival, and cancer recurrence rates in the Iran National Cancer Registry (INPCR). Future studies should address these gaps through cohort studies or data linkage approaches. Additionally, mortality statistics should be explored alongside incidence statistics for a comprehensive understanding. Further research focusing on factors related to cancers through ecological studies is recommended. Lastly, the data only extend to 2016, and the current cancer incidence scenario in 2023 may differ.

## 5 CONCLUSION

The study findings disclose the updated rankings of cancers in both men and women for the year 2016. Notably, prostate cancer has emerged as the most prevalent cancer among men, a shift from its ranking in 2014. The majority of cancer types exhibit an increase when compared to previous years. Potential explanations for these variations between the 2014 and 2016 data include enhancements in the registration and reporting system, improved cancer diagnosis methodologies, heightened accuracy in data collection, and an overall rise in the number of reported cancer cases.

## AUTHOR CONTRIBUTIONS

**Fatemeh Hadavandsiri**: Developed the conceptual framework and verified the analytical methods, performed the analytic calculations and interpretation and wrote original draft preparation, review & editing. **Hamid Salehiniya**: Review & editing. **Yekta Rahimi**: Writing—review & editing. **Leila Allahqoli**: Writing—review & editing. **Ehsan Ghazanfari Savadkoohi**: Writing. **Mohammad Esmaeil Akbari**: Review & editing.

## CONFLICT OF INTEREST STATEMENT

The authors have stated explicitly that there are no conflicts of interest in connection with this article.

## ETHICS STATEMENT

The ethical approval was granted from the ethics committee and review board of the Cancer Research Center, Shahid Beheshti University of Medical Sciences (SBMU), Tehran, Iran (IR.SBMU.CRC.REC.1401.003).

## Data Availability

The datasets of the current study are not publicly available and the data is available only on reasonable request to the corresponding author.

## References

[1] Quaresma M , Coleman MP , Rachet B . 40‐year trends in an index of survival for all cancers combined and survival adjusted for age and sex for each cancer in England and Wales, 1971‐2011: a population‐based study. Lancet (London, England). 2015;385(9974):1206‐1218. 25479696 10.1016/S0140-6736(14)61396-9

[2] Collaboration GBoDC . Global, regional, and national cancer incidence, mortality, years of life lost, years lived with disability, and disability‐adjusted life‐years for 29 cancer groups, 1990 to 2016: a systematic analysis for the global burden of disease study. JAMA Oncol. 2018;4(11):1553‐1568. 29860482 10.1001/jamaoncol.2018.2706 PMC6248091

[3] Dolatkhah R , Somi MH , Kermani IA , et al. Increased colorectal cancer incidence in Iran: a systematic review and meta‐analysis. BMC Public Health. 2015;15:997. 26423906 10.1186/s12889-015-2342-9 PMC4589975

[4] Ferlay J , Ervik M , Lam F , et al. Global Cancer Observatory: Cancer Today. International Agency for Research on Cancer; 2018. Accessed 4/02/2021. https://gco.iarc.fr/today

[5] Mousavi SM , Gouya MM , Ramazani R , Davanlou M , Hajsadeghi N , Seddighi Z . Cancer incidence and mortality in Iran. Ann Oncol. 2009;20(3):556‐563. 19073863 10.1093/annonc/mdn642

[6] UK CR . *Worldwide Cancer Incidence Statistics*. Updated 27 March 2023. https://www.cancerresearchuk.org/health-professional/cancer-statistics/worldwide-cancer/incidence#heading-One

[7] Roshandel G , Ferlay J , Ghanbari‐Motlagh A , et al. Cancer in Iran 2008 to 2025: recent incidence trends and short‐term predictions of the future burden. Int J Cancer. 2021;149(3):594‐605. 33884608 10.1002/ijc.33574

[8] Mohebbi E , Nahvijou A , Hadji M , et al. Iran cancer statistics in 2012 and projection of cancer incidence by 2035. Basic Clin Cancer Res. 2017;9(3):3‐22.

[9] Roshandel G , Ghanbari‐Motlagh A , Partovipour E , et al. Cancer incidence in Iran in 2014: results of the Iranian National Population‐based Cancer Registry. Cancer Epidemiol. 2019;61:50‐58. 31132560 10.1016/j.canep.2019.05.009

[10] Rafiemanesh H , Rajaei‐Behbahani N , Khani Y , et al. Incidence trend and epidemiology of common cancers in the center of Iran. Global J Health Sci. 2015;8(3):146‐155. 10.5539/gjhs.v8n3p146 PMC4804019 26493417

[11] Sheikh M , Poustchi H , Pourshams A , et al. Individual and combined effects of environmental risk factors for esophageal cancer based on results from the Golestan cohort study. Gastroenterology. 2019;156(5):1416‐1427. 30611753 10.1053/j.gastro.2018.12.024 PMC7507680

[12] Etemadi A , Abnet CC , Kamangar F , et al. Impact of body size and physical activity during adolescence and adult life on overall and cause‐specific mortality in a large cohort study from Iran. Eur J Epidemiol. 2014;29(2):95‐109. 24557643 10.1007/s10654-014-9883-6 PMC5761059

[13] Kassa M , Grace J . The global burden and perspectives on non‐communicable diseases (NCDs) and the prevention, data availability and systems approach of NCDs in low‐resource countries. Public Health in Developing Countries‐Challenges and Opportunities. IntechOpen; 2019.

[14] Almasi Z , Rafiemanesh H , Salehiniya H . Epidemiology characteristics and trends of incidence and morphology of stomach cancer in Iran. Asian Pac J Cancer Prev. 2015;16(7):2757‐2761. 25854359 10.7314/apjcp.2015.16.7.2757

[15] Talaiezadeh A , Tabesh H , Sattari A , Ebrahimi S . Cancer incidence in southwest of Iran: first report from Khuzestan population‐based cancer registry, 2002‐2009. Asian Pac J Cancer Prev. 2013;14(12):7517‐7522. 24460327 10.7314/apjcp.2013.14.12.7517

[16] Rohani‐Rasaf M , Abdollahi M , Jazayeri S , Kalantari N , Asadi‐Lari M . Correlation of cancer incidence with diet, smoking and socio‐economic position across 22 districts of Tehran in 2008. Asian Pac J Cancer Prev. 2013;14(3):1669‐1676. 23679254 10.7314/apjcp.2013.14.3.1669

[17] Fitzmaurice C , Abate D , Abbasi N , et al. Global, regional, and national cancer incidence, mortality, years of life lost, years lived with disability, and disability‐adjusted life‐years for 29 cancer groups, 1990 to 2017: a systematic analysis for the global burden of disease study. JAMA Oncol. 2019;5(12):1749‐1768. 31560378 10.1001/jamaoncol.2019.2996 PMC6777271

[18] Wei W , Zeng H , Zheng R , et al. Cancer registration in China and its role in cancer prevention and control. Lancet Oncol. 2020;21(7):e342‐e349. 32615118 10.1016/S1470-2045(20)30073-5

[19] Iran SCo . *Iran Statistical Yearbook*; 2022. https://www.amar.org.ir/english/Iran-Statistical-Yearbook/Statistical-Yearbook-2016-2017

[20] Jack A , Percy CL , Sobin L , Whelan S . International Classification of Diseases for Oncology: ICD‐O. World Health Organization; 2000.

[21] Torre LA , Siegel RL , Ward EM , Jemal A . Global cancer incidence and mortality rates and trends—An UpdateGlobal cancer rates and trends—An update. Cancer Epidemiol Biomarkers Prev. 2016;25(1):16‐27. 26667886 10.1158/1055-9965.EPI-15-0578

[22] Ferlay J , Soerjomataram I , Dikshit R , et al. Cancer incidence and mortality worldwide: sources, methods and major patterns in GLOBOCAN 2012. Int J Cancer. 2015;136(5):E359‐E386. 25220842 10.1002/ijc.29210

[23] Bray F , Ferlay J , Soerjomataram I , Siegel RL , Torre LA , Jemal A . Global cancer statistics 2018: GLOBOCAN estimates of incidence and mortality worldwide for 36 cancers in 185 countries. CA Cancer J Clin. 2018;68(6):394‐424. 30207593 10.3322/caac.21492

[24] International WCRF . *Cancer Trends – Stomach Cancer Statistics*; 2023. https://www.wcrf.org/cancer-trends/stomach-cancer-statistics/

[25] Moradian F , Fararouei M , Karami M , et al. Trend of geographical distribution of stomach cancer in Iran from 2004 to 2014. BMC Gastroenterol. 2022;22(1):4. 34983394 10.1186/s12876-021-02066-z PMC8725466

[26] Ferlay J , Shin HR , Bray F , Forman D , Mathers C , Parkin DM . Estimates of worldwide burden of cancer in 2008: GLOBOCAN 2008. Int J Cancer. 2010;127(12):2893‐2917. 21351269 10.1002/ijc.25516

[27] Pakzad R , Mohammadian‐Hafshejani A , Ghoncheh M , Pakzad I , Salehiniya H . The incidence and mortality of prostate cancer and its relationship with development in Asia. Prostate Int. 2015;3(4):135‐140. 26779461 10.1016/j.prnil.2015.09.001 PMC4685206

[28] Lei S , Zheng R , Zhang S , et al. Global patterns of breast cancer incidence and mortality: a population‐based cancer registry data analysis from 2000 to 2020. Cancer Commun. 2021;41(11):1183‐1194. 10.1002/cac2.12207 PMC8626596 34399040

[29] Aryannejad A , Saeedi Moghaddam S , Mashinchi B , et al. National and subnational burden of female and male breast cancer and risk factors in Iran from 1990 to 2019: results from the global burden of disease study 2019. Breast Cancer Res. 2023;25(1):1‐26. 37101247 10.1186/s13058-023-01633-4 PMC10131337

[30] Ataeinia B , Saeedi Moghaddam S , Shabani M , et al. National and subnational incidence, mortality, and years of life lost due to breast cancer in Iran: trends and age‐period‐cohort analysis since 1990. Front Oncol. 2021;11:561376. 33842306 10.3389/fonc.2021.561376 PMC8027299

[31] Farhood B , Geraily G , Alizadeh A . Incidence and mortality of various cancers in Iran and compare to other countries: a review article. Iran J Public Health. 2018;47(3):309. 29845017 PMC5971166

[32] Zaheer S , Shah N , Maqbool SA , Soomro NM . Estimates of past and future time trends in age‐specific breast cancer incidence among women in Karachi, Pakistan: 2004–2025. BMC Public Health. 2019;19:1‐9. 31345204 10.1186/s12889-019-7330-z PMC6659231

[33] Rawla P , Sunkara T , Barsouk A . Epidemiology of colorectal cancer: incidence, mortality, survival, and risk factors. Gastroenterol Rev/Przegląd Gastroenterologiczny. 2019;14(2):89‐103. 10.5114/pg.2018.81072 PMC6791134 31616522

[34] Rogers CR , Moore JX , Qeadan F , Gu LY , Huntington MS , Holowatyj AN . Examining factors underlying geographic disparities in early‐onset colorectal cancer survival among men in the United States. Am J Cancer Res. 2020;10(5):1592. 32509399 PMC7269786

[35] Ireland MJ , March S , Crawford‐Williams F , et al. A systematic review of geographical differences in management and outcomes for colorectal cancer in Australia. BMC Cancer. 2017;17:1‐12. 28152983 10.1186/s12885-017-3067-1 PMC5290650

[36] Safavi A , Azizi F , Jafari R , Chaibakhsh S , Safavi AA . Thyroid cancer epidemiology in Iran: a time trend study. Asian Pac J Cancer Prev. 2016;17(1):407‐412. 26838247 10.7314/apjcp.2016.17.1.407

[37] Azangou‐Khyavy M , Saeedi Moghaddam S , Rezaei N , et al. National, sub‐national, and risk‐attributed burden of thyroid cancer in Iran from 1990 to 2019. Sci Rep. 2022;12(1):13231. 35918489 10.1038/s41598-022-17115-0 PMC9346133

[38] Li M , Dal Maso L , Vaccarella S . Global trends in thyroid cancer incidence and the impact of overdiagnosis. Lancet Diab Endocrinol. 2020;8(6):468‐470. 10.1016/S2213-8587(20)30115-7 32445733

[39] Álvaro JR , Fraile BB , Torre EM , Ardanaz E , Guevara M , Apiñániz EA . Increased incidence of thyroid cancer in Navarra (Spain). Evolution and clinical characteristics, 1986–2010. Endocrinol Diab Nutr (English Ed). 2017;64(6):303‐309. 10.1016/j.endinu.2017.02.013 28604340

[40] Moleti M , Sturniolo G , Di Mauro M , Russo M , Vermiglio F . Female reproductive factors and differentiated thyroid cancer. Front Endocrinol. 2017;8:111. 10.3389/fendo.2017.00111 PMC5440523 28588554

